# Physiological analysis reveals the mechanism of accelerated growth recovery for rice seedlings by nitrogen application after low temperature stress

**DOI:** 10.3389/fpls.2023.1133592

**Published:** 2023-02-16

**Authors:** Hui Wang, Lei Zhong, Xiaoquan Fu, Shiying Huang, Desheng Zhao, Haohua He, Xiaorong Chen

**Affiliations:** ^1^ Key Laboratory of Crop Physiology, Ecology and Genetic Breeding, Ministry of Education, Jiangxi Agricultural University, Nanchang, China; ^2^ Jiangxi Super Rice Engineering Technology Center, Jiangxi Agricultural University, Nanchang, China

**Keywords:** low temperature, N application, rice seedling, growth recovery, antioxidant enzymes, endogenous hormones

## Abstract

Low temperature and overcast rain are harmful to directly seeding early rice, it can hinder rice growth and lower rice biomass during the seedling stage, which in turn lowers rice yield. Farmers usually use N to help rice recuperate after stress and minimize losses. However, the effect of N application on the growth recovery for rice seedlings after such low temperature stress and its associated physiological changes remain unclearly. Two temperature settings and four post-stress N application levels were used in a bucket experiment to compare B116 (strong growth recovery after stress) with B144 (weak growth recovery). The results showed that the stress (average daily temperature at 12°C for 4 days) inhibited the growth of rice seedlings. Compared to the zero N group, the N application group’s seedling height, fresh weight and dry weight significantly increased after 12 days. In particular, the increases in all three growth indicators were relatively higher than that of N application at normal temperature, indicating the importance of N application to rice seedlings after low temperature stress. The antioxidant enzyme activity of rice seedlings increased significantly after N application, which reduced the damaging effect of ROS (reactive oxygen species) to rice seedlings. At the same time, the soluble protein content of seedlings showed a slow decrease, while the H_2_O_2_ and MDA (malondialdehyde) content decreased significantly. Nitrogen could also promote nitrogen uptake and utilization by increasing the expression of genes related to ${\text{NH}}_{4}^{+}$ and ${\text{NO}}_{3}^{-}$ uptake and transport, as well as improving the activity of NR (nitrate reductase) and GS (glutamine synthetase) in rice. N could affect GA_3_ (gibberellin A3) and ABA (abscisic acid) levels by regulating the anabolism of GA_3_ and ABA. The N application group maintained high ABA levels as well as low GA_3_ levels from day 0 to day 6, and high GA_3_ levels as well as low ABA levels from day 6 to day 12. The two rice varieties showed obvious characteristics of accelerated growth recovery and positive physiological changes by nitrogen application after stress, while B116 generally showed more obvious growth recovery and stronger growth-related physiological reaction than that of B144. The N application of 40 kg hm^-2^ was more conducive to the rapid recovery of rice growth after stress. The above results indicated that appropriate N application promoted rice seedling growth recovery after low temperature stress mainly by increasing the activities of antioxidant enzymes and nitrogen metabolizing enzymes as well as regulating the levels of GA_3_ and ABA. The results of this study will provide a reference for the regulation of N on the recovery of rice seedling growth after low temperature and weak light stress.

## 1 Introduction

Rice (*Oryza sativa L.*) has a wide range of cultivation. It feeds more than half of the world’s population as a food crop (Ma et al., 2015). It comes from tropical and subtropical regions as a thermophilic crop with sensitivity to low temperature stress. (Huang et al., 2013a; Luo et al., 2021). Low temperature issues are prevalent in the most of important rice-growing regions in the world, such as the United States, Japan, Korea and China, etc. The development of mechanized direct seeding has also further increased the likelihood of rice experiencing low temperatures. (Hussain et al., 2016; Luo et al., 2019). In China’s double-season rice regions, early rice frequently experiences “late spring coldness” during seedling stage. It frequently occurs in combination with cold temperatures, cloudy weather and rain, seriously harming direct-seeded early rice seedling. As a result of “late spring coldness”, rice seedlings usually have poor growth, reduced tillering capacity, longer reproductive period and lower yield (Hiroyuki et al., 2007; Victoria et al., 2011; Ahmed et al., 2021). Early rice is generally sown earlier due to global warming, but the frequency of low temperature events has not decreased. In addition, global climate changes have increased the frequency of unusual weather (Tao et al., 2013; Zhang et al., 2016a). The possibility of direct seeded early rice experiencing “late spring coldness” weather during prophase stage is also increasing. Slow growth and reduced yield of rice due to low temperature has been an important concern for researchers (Liu et al., 2013; Wang et al., 2016).

It is well known that low temperature stress frequently leads to excessive accumulation of reactive oxygen species in the plant. This causes disruption of cell membrane integrity and abnormalities in physiological metabolic processes, which in turn leads to cell death (Airaki et al., 2012; Obata and Fernie, 2012; Huang et al., 2013b; Cui et al., 2018). Antioxidant enzymes such as SOD (superoxide dismutase), POD (peroxidase), and CAT (catalase) play an important role in plant stress protection, and they can efficiently scavenge reactive oxygen species and reduce plant damage caused by stress (Zhang et al., 2016b; Shi et al., 2020). In addition, low temperatures also substantially reduce the nitrogen uptake capacity of rice, while the uptake and utilization of nitrogen by rice is directly related to their yield (Shimono et al., 2012; Zhou et al., 2018). The researchers analyzed temperature data from 1961 to 2018 from weather stations in Jiangxi Province of China. They found that because of global warming, the frequency of extreme cold weather such as severe “late spring coldness” appeared to decrease, but the frequency of mild and moderate “late spring coldness” increased (Guo et al., 2021). It can be seen that early rice in Jiangxi province and other double-season rice areas in southern China is mainly facing mild and moderate “late spring coldness” weather, and the resulting stunted growth of rice seedlings. Direct seeding of rice, in contrast to transplanting of seedlings, results in changes in the composition and density of weeds, and too many weeds can harm the development and growth of rice and even result in significant yield losses (Khaled et al., 2020; He et al., 2022). Low temperatures cause growth stunting and weakness for rice, especially weeds have a significant competitive advantage, and the application of herbicides is vulnerable to serious damage to rice seedlings (Van Bruggen et al., 2018). It has also been reported that low temperatures reduce the activity of membrane-bound enzymes, inhibit herbicide metabolism, and increase herbicide damage to rice (Martini et al., 2015). Therefore, rapid growth recovery of rice after low temperature stress is important to accelerate growth and development process, suppress weed growth, and reduce yield loss.

In recent years, the regulating function of N in crops’ reactions to external stresses has been increasingly explored. N application mainly affects plant stress resistance by regulating plant uptake and distribution of nitrogen (Du et al., 2020). Proper supplementation of N fertilization enhances the antioxidant capacity of crops and reduces the negative effects caused by stress (Thiem et al., 2014; Zhong et al., 2017). It was also discovered that N supplementation after low temperature stress could promote the growth of rice tillers, while the number of tillers increased was closely related to the increase in nitrogen accumulation (Peng et al., 2015). When rice is exposed to low temperatures, a higher N application is effective in promoting nitrogen uptake, tillering, and the development of leaf area in rice (Zhou et al., 2018). It is easy to see that most of the current studies have focused on the regulatory effects of N application after low temperature stress on the yield aspects of rice. Liu indicated that N as well as zinc could promote the growth of rice tillers after low temperature and enhance the resistance of rice to stress (Liu et al., 2019; Liu et al., 2022). However, it is mainly for the slow temperature rise and long low temperature period in spring in Northeast China, the varieties they used were also japonica rice varieties selected for cold tolerance. The “late spring coldness” weather that direct-seeded early rice (indica) endured in the middle and lower reaches of the Yangtze River of China is very different from this. The weather for the region is generally characterized by a rapid drop in temperature followed by a rapid rise, and is usually accompanied by rainy and cloudy. Therefore, the regulation effect and its mechanism for compensatory growth of N on direct-seeded early rice seedlings in southern China after low temperature and weak light stress need to be further investigated.

It has been suggested that plants usually undergo compensatory growth after short-term stress and that moderate stress helps plants to adapt to the external environment (Acevedo et al., 1971; Avramova et al., 2015). In our previous experiments, we found significant differences in the growth recovery ability of different early rice varieties after low temperature and weak light stress when simulating “late spring coldness” weather. Accordingly, we conducted screening tests on 131 early rice varieties and investigated the mechanism of differences between strong and weak growth recovery materials (Wang et al., 2022a; Wang et al., 2023). In order to investigate the effects of supplemental nitrogen fertilization on the growth indexes, osmoregulatory substances, nitrogen accumulation, related enzyme activities, and endogenous hormones of rice after the stress, we chose two materials with clearly different growth recovery abilities after the stress and conducted a bucket experiment in an artificial climate chamber with different N concentration treatments. Our hypothesis is that the stress would inhibit the growth of rice seedlings. N application after temperature recovery would compensate for this negative effect to some extent and be reflected in plant height and matter accumulation, as well as beneficial changes in seedling antioxidant enzyme activity, nitrogen metabolizing enzyme activity, N accumulation and endogenous hormone content. The main objectives of this study were: 1. Determining the regulatory effect and mechanism of N on growth recovery of early rice after the stress; 2. Investigating the differences in the regulatory effect of N on early rice with different growth recovery ability after the stress; 3. Determining the appropriate amount of N after the stress. The results of the study can provide a reference for the nitrogen application in early rice after low temperature and weak light stress.

## 2 Materials and methods

### 2.1 Plant materials and growth conditions

B116 (R310/R974, F_17_; strong compensatory growth type after low temperature stress) and B144 (Zaoxian ST66-3; weakly compensatory growth type after low temperature stress) (Wang et al., 2023) were selected for bucket experiment. The experiment was conducted in July 2022 in an artificial climate chamber at the Crop Physiology, Ecology and Genetic Breeding Laboratory, Jiangxi Agricultural University, Nanchang, Jiangxi Province, China (115°50′E, 28°46′N). The bucket used had an upper internal diameter of 29.0 cm, a bottom internal diameter of 23.5 cm, and a height of 24.0 cm. The soil for the experiment was taken from the shallow layer (0-20 cm) of the paddy field and it was placed in the courtyard of the crop genetic breeding experiment base of Jiangxi Agricultural University to dry naturally. After drying, the soil was crushed with FT-1000A soil crusher (Shandong AMBER Instrument Co., Ltd.) and filtered using 100 mesh sieve. Soil pH was 5.4, organic matter content was 32.8 g kg^-1^, total nitrogen content was 0.174%, and effective nitrogen, phosphorus and potassium contents were 113.0, 17.3 and 108.4 mg kg^-1^, respectively. Each bucket was filled with 10 kg of air-dried soil, and 4 g of compound fertilizer (pure nitrogen-P_2_O_5_-K_2_O = 15%-15%-15%) was applied as a base fertilizer, and the soil used was soaked in water for 5 days before the start of the experiment.

### 2.2 Experimental design

Uniform and full seeds with high germination rate was selected and disinfected with 3% sodium hypochlorite solution before the start of the experiment, and they were rinsed with water and soaked in sprouting bags. About 15 seeds per bucket were sown after they were dew white.

The sowing date was July 22, 2022, and the experiment was a randomized design with three types of factors: 1) temperature and light - the control group (CK and N) had an average daily temperature of 26°C and 600 μmolm^-2^s^-1^ of effective photosynthetic radiation. The low temperature and nitrogen application group (LN) had an average daily temperature of 12°C and 300 μmolm^-2^s^-1^ of effective photosynthetic radiation; 2) Variety - strong compensatory growth material B116 and weak compensatory growth material B144; 3) N application after the stress- Urea was used as a nitrogen source of four levels, LN0 (0 kg hm^-2^), LN20 (0.5 g per bucket, equivalent to 20 kg hm^-2^), LN40 (1 g per bucket, equivalent to 40 kg hm^-2^), and LN80 (2 g per bucket, equivalent to 80 kg hm^-2^) (Xiong et al., 2018; Liu et al., 2019). Rice seedlings in CK also received four levels of N treatment (CK, 0 g per bucket; N20, 0.5 g per bucket; N40, 1 g per bucket; N80, 2 g per bucket) on the same day that the low temperature and weak light stress ended. The experiment consisted of 16 treatments, with five biological replicates for each treatment. Treatments were started when the rice seedlings grew to the first leaf stage (July 27). The test conditions were referred to the national standard for inverse temperature meteorological indicators of the People’s Republic of China^1^ and the standard for “late spring coldness” in Jiangxi Province (Guo et al., 2021). CK and N was set with a photoperiod of 12 h, 600 μmolm^-2^s^-1^ of effective photosynthetic radiation, daytime (6:00 to 18:00) and nighttime (18:00 to 6:00) temperatures of 27°C and 25°C, respectively, and relative humidity of 75%. The LN photoperiod was 12 h, the daytime (6:00 to 18:00) and nighttime (18:00 to 6:00) temperatures were 14°C and 10°C, respectively, and the effective photosynthetic radiation was set at 300 μmolm^-2^s^-1^ (50% of CK and N) for 4 days, the relative humidity was set at 75%. The first sampling was on the last day of the treatment, recorded as day 0. After sampling, urea was dissolved in water and applied, and set the temperature and light of LN was set to be the same as CK and N. And subsequent sampling surveys were conducted at day 3, 6, 9 and 12.

### 2.3 Investigation of rice seedling growth indicators

In order to investigate seedling height, fresh weight, and dry weight, data were gathered every three days for a total of five times starting on day 0. Rice seedlings of uniform growth were selected for measuring plant height and then sampled. The rice seedlings were washed and dried on filter paper and placed in an oven at 37°C for 5 min for fresh weight measurement and then in an oven at 80°C for dry weight measurement. 5 biological replicates were performed for each treatment.

### 2.4 Determination of soluble protein, MDA, H_2_O_2_ content and antioxidant enzyme activity

Soluble protein, MDA, H_2_O_2_ content and antioxidant enzyme activity were measured on day 0, day 3, day 6, day 9 and day 12 after stress. Uniformly grown rice seedlings were selected and sampled with leaf tissue, after which they were washed with distilled water, immediately placed in liquid nitrogen and frozen, then stored in a -80°C refrigerator. A total of three biological replicates were performed. The above indexes were measured using Bradford Protein Assay Kit from Beyotime Biotechnology Co., Ltd. (Shanghai, China), MDA-1-Y assay kit, H_2_O_2_-1-Y assay kit, SOD assay kit, CAT assay kit and POD assay kit (Comin Biotechnology Co. Ltd., Suzhou, China), respectively.

### 2.5 Determination of total nitrogen content and NR, GS activity

Uniformly grown rice seedlings were selected and sampled with leaf tissue, after which they were washed with distilled water, immediately placed in liquid nitrogen and frozen, then stored in a -80°C refrigerator. A total of three biological replicates were performed. NR and GS activities were measured using NR-2-W, GS-2-Y assay kits (Comin Biotechnology Co. Ltd., Suzhou, China), respectively. The dried samples on day 12 were crushed and analyzed with a Dumas nitrogen determinator, and the total nitrogen content of the individual plants was calculated from the N content of the samples as well as the dry weight of the rice seedlings.

### 2.6 Determination of endogenous hormone content

The endogenous hormone concentrations of GA_3_ and ABA in the leaves of the above samples were determined by high performance liquid chromatography according to the instructions of a commercial kit (Comin Biotechnology Co., Ltd., Suzhou, China).

### 2.7 RNA Extraction and RT-qPCR

Uniformly grown rice seedlings were selected and sampled with whole rice seedlings, after which they were washed with distilled water and were immediately placed in liquid nitrogen and frozen, then stored in a -80°C refrigerator. Total RNA from leaves and roots were extracted using Trizole method (RNAiso Plus, Code No. 9109, Takara, Japan). First-strand complementary DNA (cDNA) synthesis was performed on 1 μg of total RNA using the PrimeScript 1st Strand cDNA Synthesis Kit (TaKaRa, Japan). RT-qPCR was performed with the Light Cycler 480 SYBR Green 1 Master (Roche, Switzerland) on a Light Cycler 480 Real-Time PCR System (Roche) according to the manufacturer’s instructions. We used *OsActin* as the internal control. The primers for RT-qPCR analysis are listed in **Table S1**.

### 2.8 Statistical analysis

The obtained data were analyzed for significance of differences using SPSS 26.0 and tabulated as well as images were plotted using Excel 2019 and GraphPad Prism 8.0. The data were analyzed through analysis of variance, and the differences among the treatments were tested using the least significant difference (LSD) test with a probability level of 0.05.

## 3 Results

### 3.1 Effect of N application on growth indicators of rice seedlings after stress

**Figure 1:**
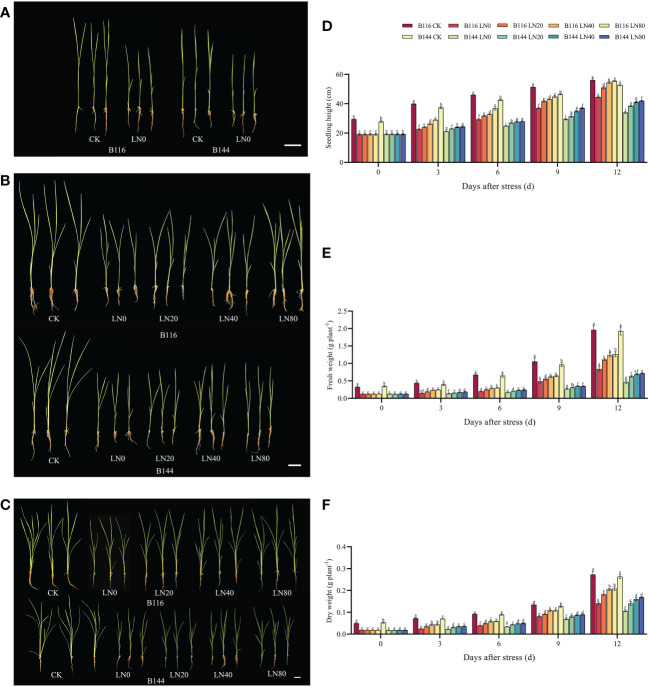
Effect of nitrogen application after low temperature and weak light stress on the growth indexes of rice seedlings at different periods. **(A–C)** show the appearance of rice seedlings at day 0, 6 and 12 after stress, respectively. **(D–F)** show the comparison of seedling height, fresh weight and dry weight for the different treatments, respectively. LN0, LN20, LN40, LN80 represent 0 kg hm^-2^, 20 kg hm^-2^, 40 kg hm^-2^ and 80 kg hm^-2^ urea application, respectively. Values are means ± standard deviations of five biological replicates, and different lowercase letters represent significant differences at p<0.05 based on Duncan’s test.

Stress significantly slowed the growth of rice seedlings, according to the analysis of growth indicators. (**Figures 1A–D**). Compared with CK, the seedling height of B116 and B144 were decreased by 34.9% and 30.7% respectively after the stress. After N application, seedling height growth recovery of rice was faster and significantly higher than that of LN0 from day 3 to day 12. With the increase of N concentration, the growth recovery of rice seedling height was accelerated, and at day 12, B116 LN40 and B116 LN80 could even approach B116 CK (97.3% and 98.9% of B116 CK, respectively). The seedlings height of B144 LN40 and B144 LN80 was only 77.9% and 79.4% that of B144 CK. Notably, no significant difference was observed between B144 LN40 and B144 LN80 at day 3 and day 6. V, N and T had significant effects on seedling height (P<0.01), while the interaction effects of two or three of them reached significant levels (P<0.01) on seedling height (**Table 1**).

**Table 1:** Results of two-way ANOVA on differences in seedling height, fresh weight and dry weight of rice after the stress.

Factors	Seedling height	Fresh weight	Dry weight
Variety (V)	< 0.001	< 0.001	< 0.001
Nitrogen (N)	< 0.001	< 0.001	< 0.001
Growth recovery time (T)	< 0.001	< 0.001	< 0.001
V*N	**	**	**
V*T	**	**	**
N*T	**	**	**
V*N*T	**	**	ns

* and ** represent P < 0.05 and P < 0.01, respectively, ns represents no significant.

Stress had an equal and even greater impact on the fresh and dry weights of rice seedlings (**Figures 1E, F**) than it did on seedling height. Compared with CK, the fresh weight of B116 and B144 was decreased by 61.4% and 64.1%, and the dry weight was decreased by 61.8% and 64.8% respectively after the stress. The two rice varieties also performed similarly to the recovery effect of seedling height after N application, but the increase in N fertilizer concentration (from LN40 to LN80) did not have a significant effect on their recovery, and only at day 12, a significant difference in fresh weight between B116 LN40 and B116 LN80 was observed. V, N, and T had significant effects on both fresh and dry weight (P<0.01), while the interaction effects of two or three of them reached highly significant levels on fresh weight (P<0.01), but the interaction of the three factors did not have significant effects on dry weight.

The nitrogen application test under normal temperature and light (N) showed that the increase of rice seedling height was proportional to the applied N concentration, but the fresh weight and dry weight of both rice seedlings under N80 condition were even lower than those under N40 (**Figure S1**). It means that the rice seedlings height was overgrowth. Meanwhile, the increase in growth indicators of rice seedlings under normal conditions by N was much lower than that of rice seedlings after the stress. At day 12, compared with CK, two rice varieties increased seedling height, fresh weight and dry weight by 0.92% to 8.25%, 3.69% to 8.61% and 0.02% to 2.36%, respectively, after N application under normal conditions. And N application after low temperature increased their seedling height, fresh weight and dry weight by 13.31% to 24.66%, 32.34% to 53.30% and 26.69% to 54.82%, respectively, compared with LN0. V, N and T had significant effects (P<0.01) on seedling height and fresh weight (**Table S2**), while N had no significant effect on dry weight. There was no significant interaction effect between V and N on growth indicators, a significant interaction effect between V and T on growth indicators, and no significant interaction effect between N and T on dry weight. There was a significant interaction effect of the three factors on seedling height only.

### 3.2 Effect of N application on soluble protein, H_2_O_2_ and MDA content of rice seedlings after stress

**Figure 2:**
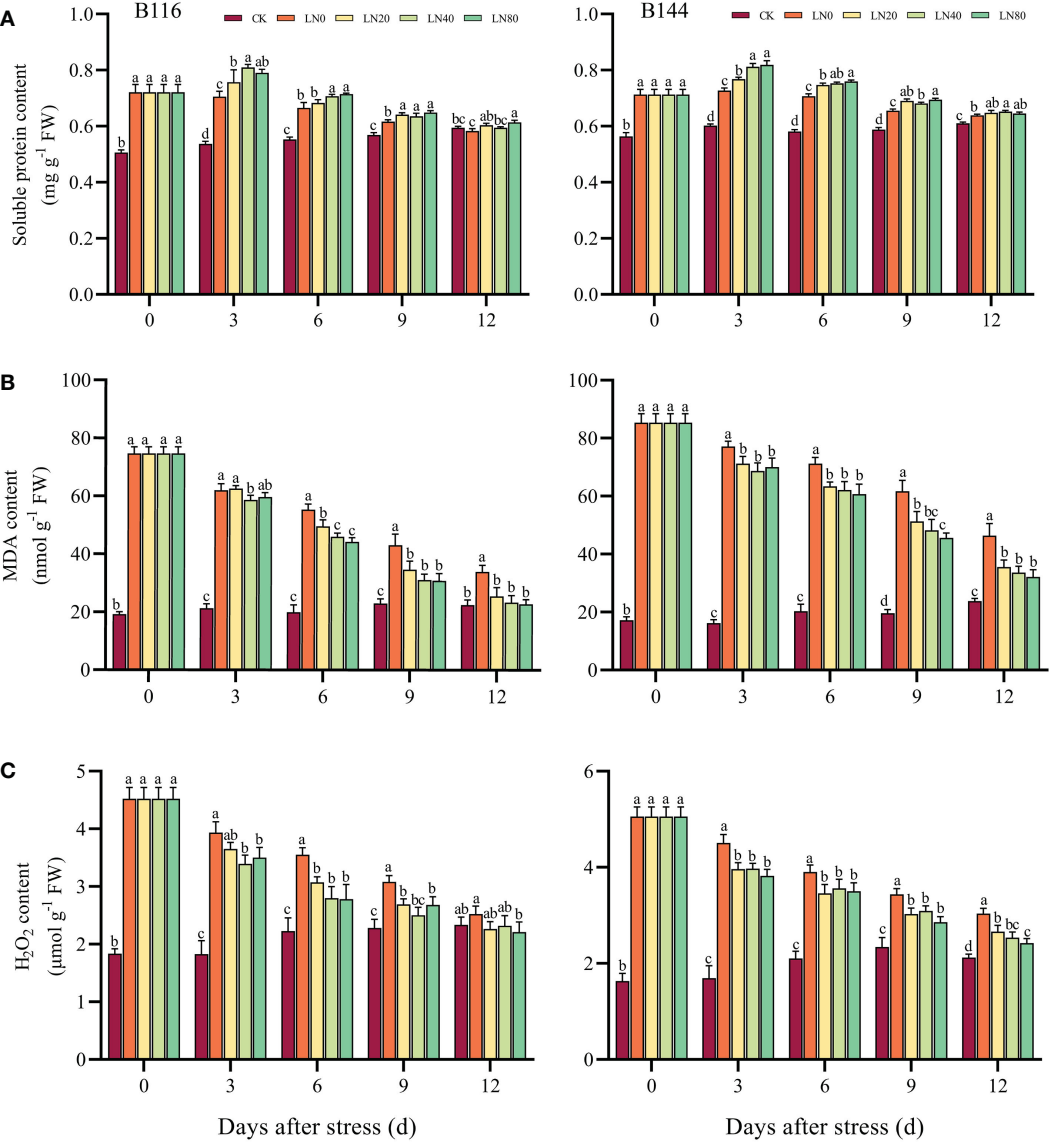
Effect of nitrogen application after low temperature and weak light stress on soluble protein content **(A)**, MDA content **(B)** and H_2_O_2_ content **(C)** of rice seedlings at different periods. LN0, LN20, LN40, LN80 represent 0 kg hm^-2^, 20 kg hm^-2^, 40 kg hm^-2^ and 80 kg hm^-2^ urea application, respectively. Values are means ± standard deviations of five biological replicates, and different lowercase letters represent significant differences at p<0.05 based on Duncan’s test.

Under stress, soluble proteins play an important role in maintaining the balance of cellular osmotic potential (Xiong et al., 2019). Following the stress, B116 and B144 had higher soluble protein contents than CK by 42.5% and 26.6%, respectively (**Figures 2**). The N application caused a significant increase in the soluble protein content of B116 and B144 from day 0 to day 6. It showed that the higher the N concentration, the higher the increase in soluble protein content. The gap between the groups gradually decreased as the recovery time increased. On day 12, the soluble protein contents of B116 LN were all close to those of CK, with the soluble protein contents of B116 LN0 being significantly lower than the CK, while those of B144 LN were still significantly higher than CK.

Excessive ROS in plants, especially H_2_O_2_, can cause excessive oxidation of important macromolecules such as DNA, proteins and membrane lipids by inhibiting ROS scavenging enzymes under stress, thereby damaging the plant (Obata and Fernie, 2012; Cui et al., 2018). Stress caused a significant increase in the H_2_O_2_ content of B116 and B144, while the application of N could accelerate their removal of H_2_O_2_, and there was no significant difference in the H_2_O_2_ content of B116 LN40 compared with CK at day 9. At day 12, B116 LN was basically close to or even lower than the CK, but the H_2_O_2_ content of B144 LN was still significantly higher than the CK.

MDA, as the end product of cell membrane lipid peroxidation, is commonly used to measure the degree of peroxidation of membrane lipids (Yang et al., 2014). After the stress, the MDA contents of both B116 and B144 increased significantly (289.9% and 396.9%, respectively), while N application contributed to the rapid reduction of rice MDA contents. As the N concentration increased, the MDA content decreased further. The effects of V, N and T on soluble protein, H_2_O_2_ and MDA content were significant (P<0.01) (**Table S3**), but only the interaction effects of V with T and N with T on soluble protein concentration and MDA concentration reached a significant level (P<0.01).

### 3.3 Effect of N application on antioxidant enzymes activity of rice seedlings after stress

**Figure 3:**
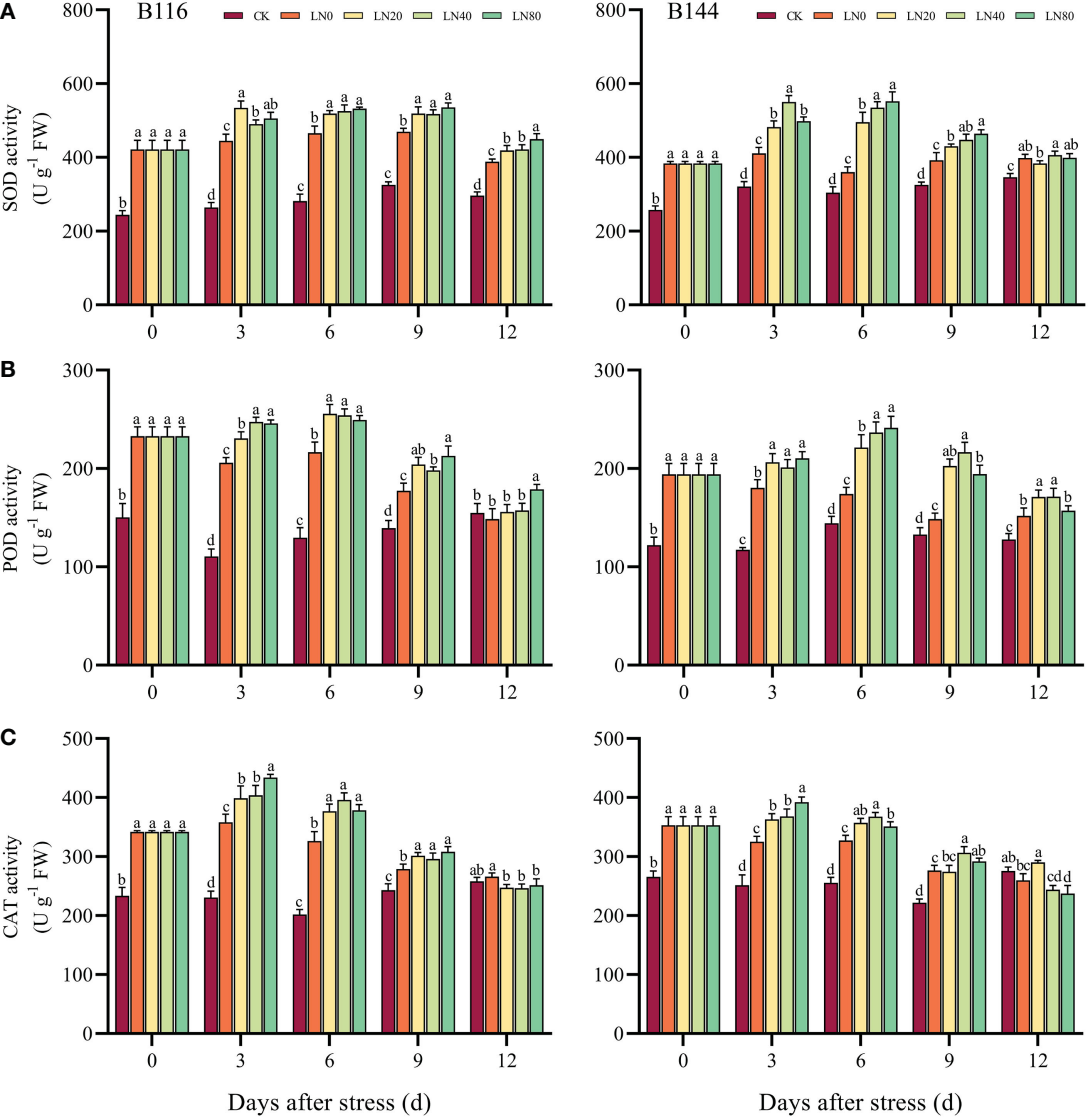
Effects of nitrogen application after low temperature and weak light stress on SOD activity **(A)**, POD activity **(B)** and CAT activity **(C)** of rice seedlings at different periods. LN0, LN20, LN40, LN80 represent 0 kg hm^-2^, 20 kg hm^-2^, 40 kg hm^-2^ and 80 kg hm^-2^ urea application, respectively. Values are means ± standard deviations of five biological replicates, and different lowercase letters represent significant differences at p<0.05 based on Duncan’s test.

When plants are stressed, large amounts of ROS accumulate rapidly in their bodies, causing serious damage to plant cells. The antioxidant enzymes can maintain the balance of ROS in plants and are important in detoxifying ROS and reducing the damage of stress to plants (Zhang et al., 2016b). After the stress, there was a significant increase in SOD, POD and CAT activities in both B116 and B144, while all three antioxidant enzyme activities increased more in B116 than in B144 compared to CK (**Figures 3A–C**). With the increase of time, the antioxidant enzyme activities of the two rice varieties showed a trend of increasing and then decreasing, similar to the trend of soluble protein concentration. Nitrogen application resulted in a significant increase in antioxidant enzyme activity in rice, but in most cases the antioxidant enzyme activity did not increase significantly with the increase in N concentration. It should be noted that the SOD and POD activities of B116 LN0 were significantly higher than those of B144 LN0 between days 0 and 9, and after N application, the SOD and POD activities of B144 were able to approach or even exceed those of B116. However, the effect of N on the regulation of CAT activity of B144 was not as obvious as that of SOD and POD. V, N, and T had significant effects (P<0.01) on the activities of SOD, POD, and CAT (**Table S3**). However, the interaction effect of V and T did not have a significant effect on the activities of POD and CAT.

### 3.4 Effect of N application on N uptake and metabolism of rice seedlings after stress

**Figure 4:**
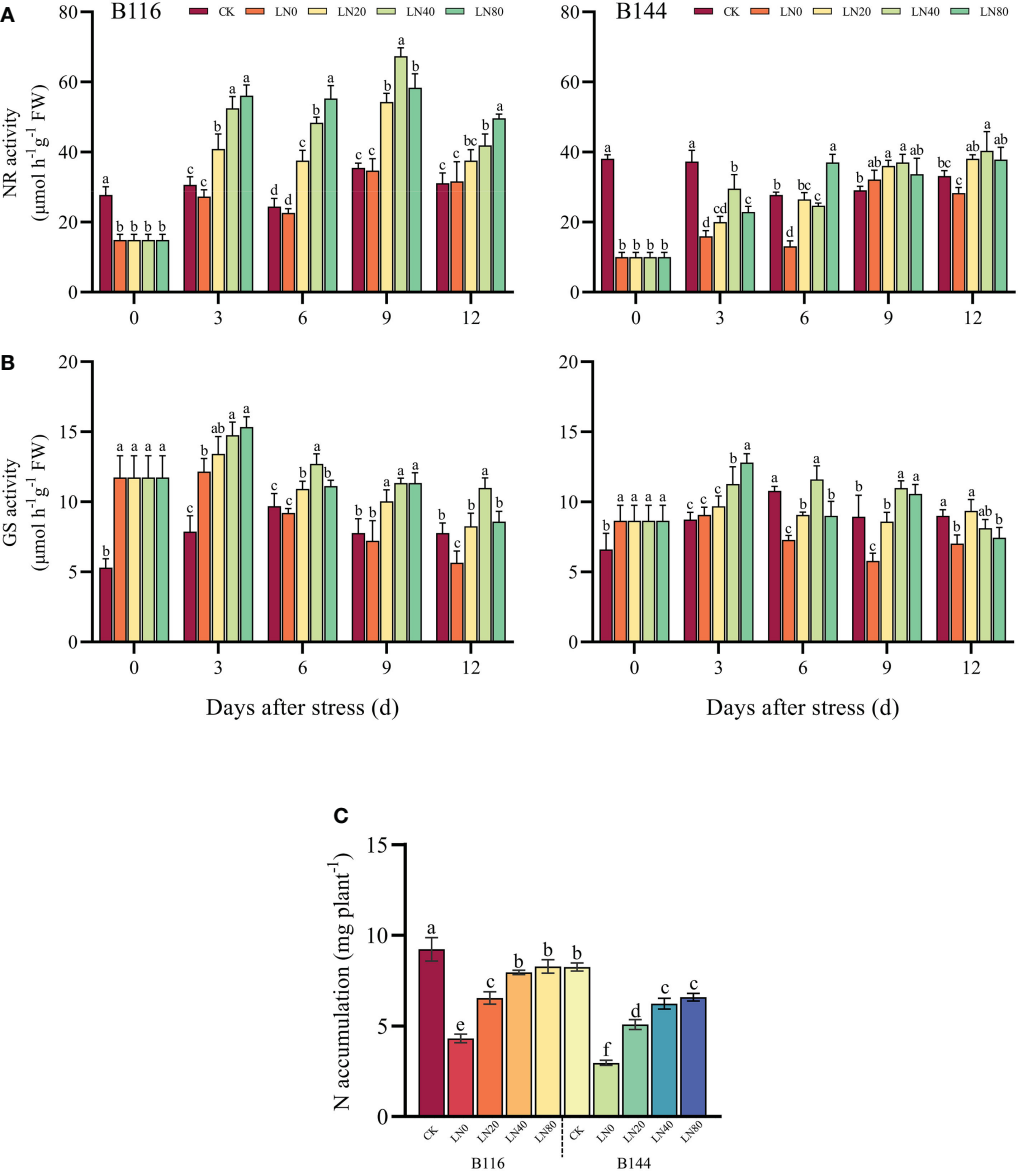
Effects of nitrogen application after low temperature and weak light stress on NR activity **(A)**, GS activity **(B)** and N accumulation **(C)** of rice seedlings at different periods. LN0, LN20, LN40, LN80 represent 0 kg hm^-2^, 20 kg hm^-2^, 40 kg hm^-2^ and 80 kg hm^-2^ urea application, respectively. Values are means ± standard deviations of five biological replicates, and different lowercase letters represent significant differences at p<0.05 based on Duncan’s test.

NR and GS are the key enzymes involved in plant N metabolism (Ohashi et al., 2015; Gao et al., 2019). As can be seen from the **Figures 4A, B**, stress had a significant effect on both NR and GS activities of the two rice seedlings, but the trends of NR and GS activities were completely opposite, with B116 and B144 showing a significant decrease in NR activity and a substantial increase in GS activity. N application significantly enhanced NR and GS activities of rice seedlings. For B116, higher N concentration implied higher NR activity during most of the growth recovery phase (except day 9). It should be noted that the NR activity of B144 did not show a similar pattern, and in most cases the NR activity of B144 LN40 was higher than that of B144 LN80. In terms of GS activity, higher N concentration likewise does not mean higher GS activity, and in most cases, LN40 has a better GS activity than LN80. In terms of GS activity, higher N concentration likewise does not mean higher GS activity, and in most cases, LN40 has a better GS activity than LN80. There were significant effects of V, N as well as T on the activities of NR and GS (P<0.01), while there was a significant interaction between the combination of all factors on the activities of NR and GS (P<0.01), except for the interaction of V and N, which had no significant effect on the activity of GS (**Table S4**).

The stress largely inhibited N uptake in rice (**Figure 4C**), and N accumulation in B116 LN0 and B144 LN0 was reduced by 53.2% and 64.0% respectively compared with CK. N application substantially promoted N accumulation in rice seedlings after the stress. For B116, LN20, LN40 and LN80 increased 51.5%, 85.3% and 91.7%, respectively, compared with LN0. While N application promoted B144 more significantly than B116, with B144 LN20, LN40 and LN80 increasing 71.2%, 110.0% and 121.7% respectively compared with LN0. There was a highly significant positive correlation between the increase in N accumulation and the recovery of rice seedling growth index after stress (**Figure S2**). There was a significant effect of V and N on N accumulation (P<0.01) (**Table S4**), but they did not have a significant interaction on N accumulation.

**Figure 5:**
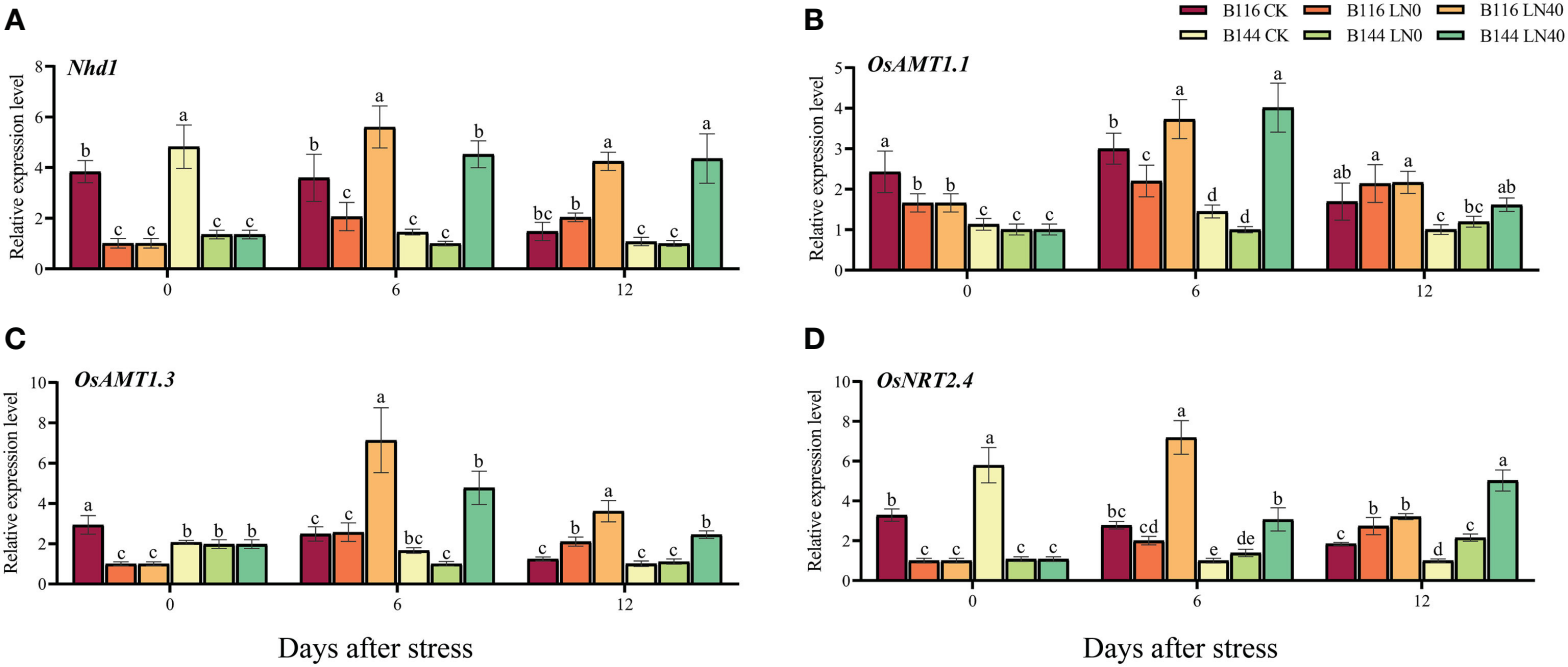
Effects of nitrogen application after low temperature and weak light stress on the relative expression levels of N uptake and transporter-related genes *Nhd1* **(A)**, *OsAMT1.1* **(B)**, *OsAMT1.3* **(C)**, and *OsNRT2.4* **(D)** of rice seedlings at different periods. LN0 and LN40 represent 0 kg hm^-2^ and 40 kg hm^-2^ urea application, respectively. Values are means ± standard deviations of three biological replicates, and different lowercase letters represent significant differences at p<0.05 based on Duncan’s test.

The results of RT-qPCR assays on genes related to ${\text{NH}}_{4}^{+}$ , ${\text{NO}}_{3}^{-}$ uptake and transport in rice showed that the stress reduced the expression of related genes (**Figure 5**). N application enhances the expression of these genes and enhances the N uptake and transport capacity of rice. This result can be corroborated with the increased N accumulation after N application.

### 3.5 Effect of N application on endogenous hormone content of rice seedlings after stress

**Figure 6:**
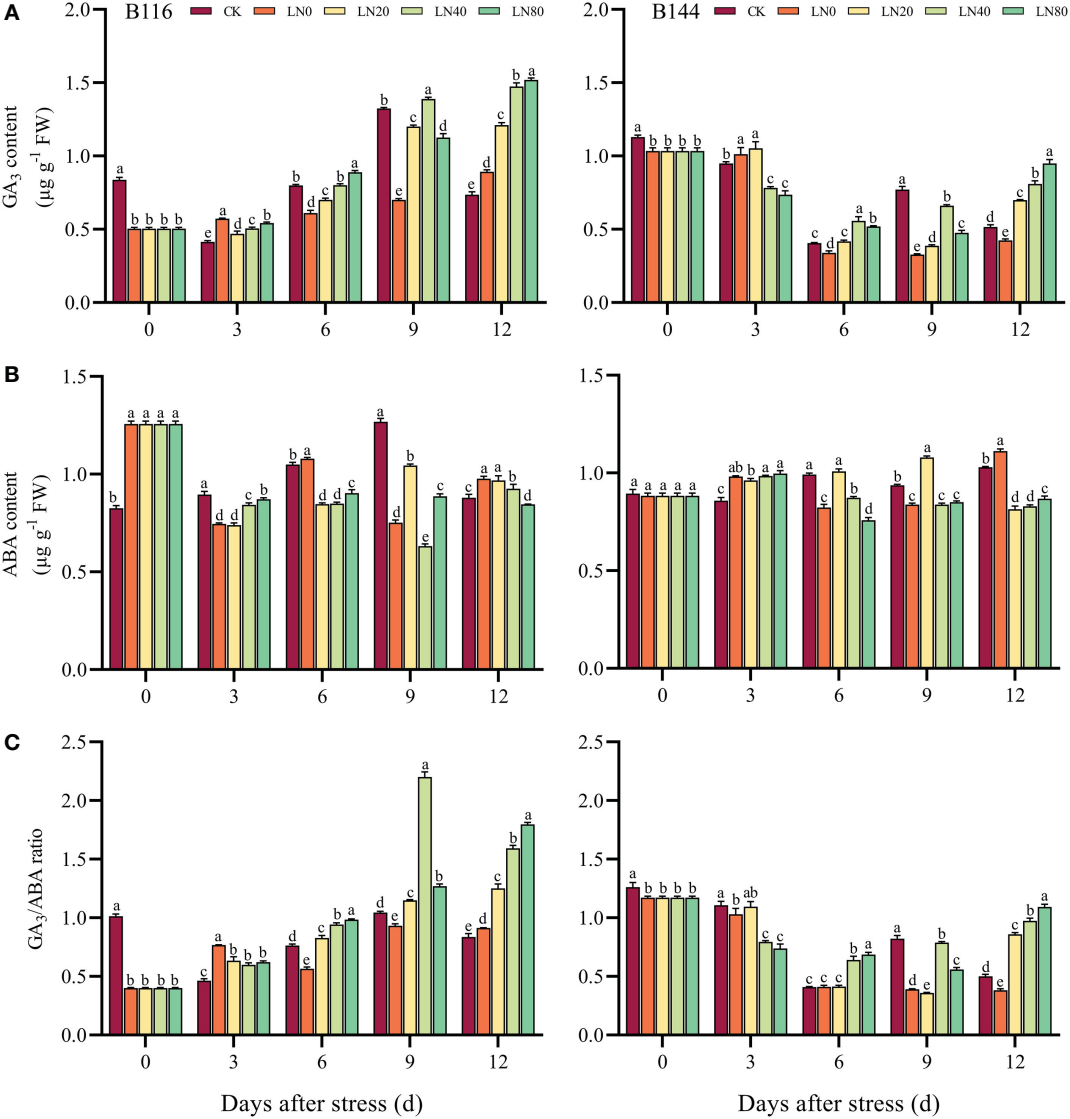
Effects of nitrogen application after low temperature and weak light stress on GA_3_ content **(A)**, ABA content **(B)** and the ratio of GA_3_ to ABA content **(C)** of rice seedlings at different periods. LN0, LN20, LN40, LN80 represent 0 kg hm^-2^, 20 kg hm^-2^, 40 kg hm^-2^ and 80 kg hm^-2^ urea application, respectively. Values are means ± standard deviations of five biological replicates, and different lowercase letters represent significant differences at p<0.05 based on Duncan’s test.

The GA_3_ content of the two rice decreased by 39.9% and 8.3% respectively after the stress compared with CK. N application can increase the GA_3_ content of rice seedlings (day 6 to day 12) (**Figure 6**). The GA_3_ content of B116 was most significantly enhanced by N at day 9, with 98.5% increase in LN40 compared with LN0, for B144 it was on day 12 that LN80 improved about 123.8% relative to LN0. Different from GA_3_, the ABA content of the two rice species produced different responses to the stress (**Figure 6C**), the ABA content of B116 was elevated by about 46.9% compared with CK and was also significantly higher than that of B144, while B144 showed a small decrease compared with CK (no significant difference). The effect of N application on ABA content in two rice varieties was also not uniform, with N application boosting ABA content in B116 in the early period (day 3) and promoting a decrease in ABA content in the later period (days 6 and 12). For B144, N application during the early period (day 3) was not effective in regulating its ABA content. At day 12, N showed a significant regulating effect on the ABA content of B144, with LN20, LN40, and LN80 containing much lower ABA content than LN0. By comparing the ratio of GA_3_ with ABA (**Figure 6C**), it can be seen that the GA_3_/ABA of both B116 and B144 decreased after stress compared with CK, with B116 showing a more pronounced decrease. N application kept GA_3_/ABA in B116 at a low level during the early period (day 3) and maintained at a high level during the later period. The trend of GA_3_/ABA changes in B144 was similar to that of B116, the difference being that there was no significant difference between B144 LN20 and B144 LN0 from day 3 to day 9. V, N, and T had significant effects (P<0.01) on GA_3_ as well as ABA content (**Table S5**), and their interaction effects on GA_3_ and ABA contents were also significant.

**Figure 7:**
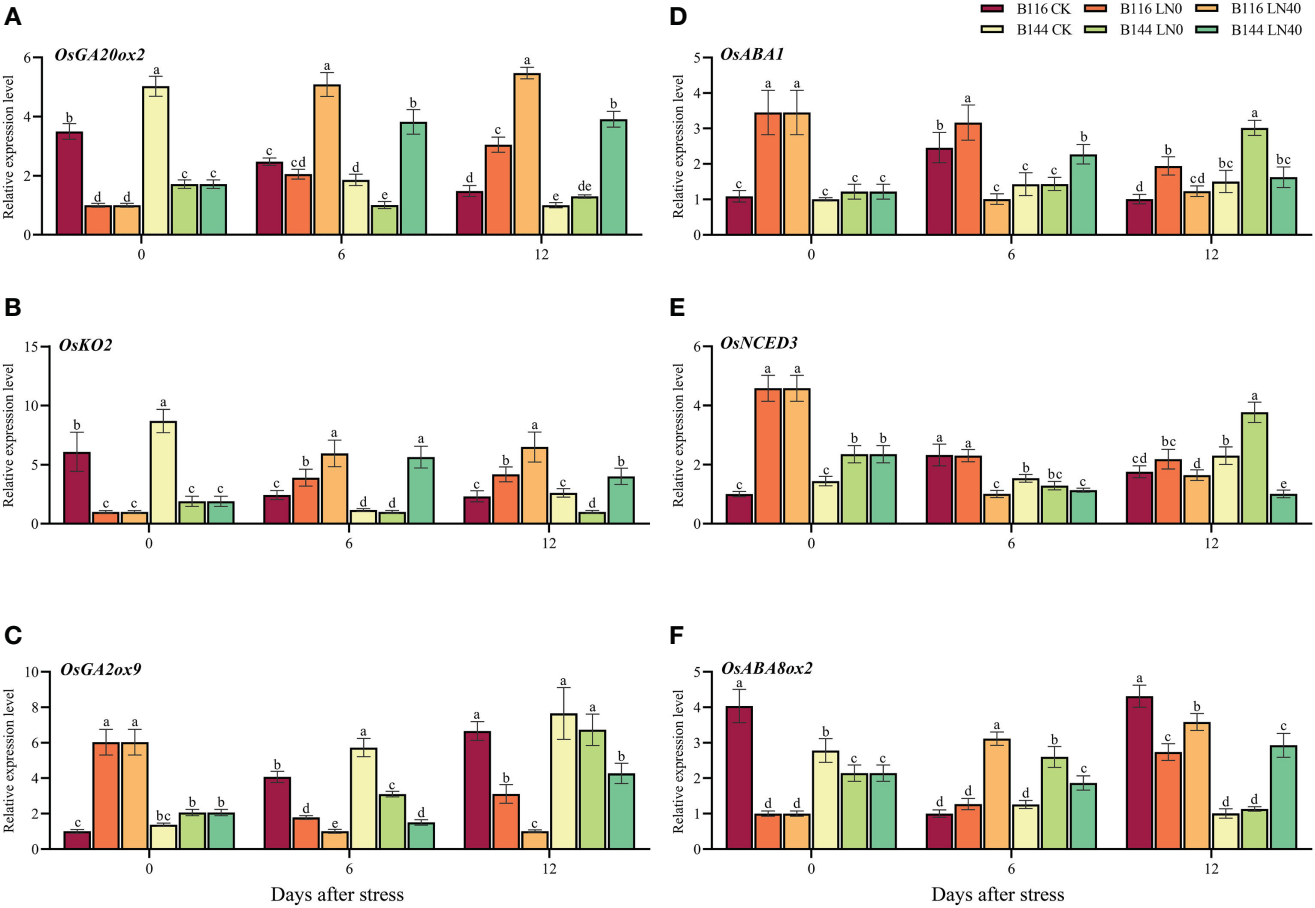
Effects of low temperature and weak stress followed by N application on the relative expression of gibberellin and ABA biosynthesis or metabolism related genes in rice seedlings at different periods. Gibberellin biosynthesis related genes *OsGA20ox2* **(A)** and *OsKO2* **(B)**; gibberellin metabolism related genes *OsGA2ox9* **(C)**; ABA biosynthesis related genes *OsABA1* **(D)** and *OsNCED3* **(E)**; ABA metabolism related genes *OsABA8ox2* **(F)**. LN0 and LN40 represent 0 kg hm^-2^ and 40 kg hm^-2^ urea application, respectively. Values are means ± standard deviations of three biological replicates, and different lowercase letters represent significant differences at p<0.05 based on Duncan’s test.

To further investigate the role of N on the regulation of GA_3_ and ABA content, we selected several key genes involved in GA_3_ and ABA biosynthesis and metabolism (**Figure 7**) for RT-qPCR assays. The results showed that the GA_3_ biosynthetic genes *OsGA20ox2*, *OsKO2* and GA_3_ metabolic gene *OsGA2ox9* showed opposite trends at day 0. The relative expression levels of *OsGA20ox2* and *OsKO2* decreased substantially compared with CK after stress, while *OsGA2ox9* showed an increasing trend. Both *OsGA20ox2* and *OsGA2ox9* decreased or increased more in B116 than in B144. N application was able to significantly increase the expression levels of *OsGA20ox2* and *OsKO2* at a later stage, while suppressing the expression of *OsGA2ox9*, which was consistent with the trend of GA_3_ content in the two rice varieties. The ABA biosynthesis genes *OsABA1* and *OsNCED3* and the ABA metabolism gene *OsABA8ox2* also showed similar trends, and N application was able to suppress the expression of *OsABA1* and *OsNCED3* and promote the expression of *OsABA8ox2* at a later stage.

## 4 Discussion

Rice, as a warm-loving crop, often experiences low temperatures when grown as early rice in the middle and lower reaches of the Yangtze River in China. This makes them grow slower and have less tiller capacity (Hiroyuki et al., 2007). The proportion of mild and moderate low temperatures in the middle and lower reaches of the Yangtze River has shown a significant increase in recent years (Velitchkova et al., 2020; Guo et al., 2021). It has been shown that the growth of rice seedlings is severely hindered when the average temperature is below 12°C (Sipaseuth et al., 2007). In our previous study, we found that strong growth recovery rice variety had stronger antioxidant capacity, nitrogen metabolism capacity and higher levels of growth promoting hormones compared to weak growth recovery variety after low temperature and weak light stress (Wang et al., 2022a). It has been shown that the rapid growth recovery of rice seedlings after stress is also closely linked to their nitrogen utilization (Liu et al., 2019). This study showed that the stress reduced the height, fresh weight and dry weight of rice seedlings. The increase in seedling height, fresh weight and dry weight of rice seedlings with N application after low temperature was much greater than that with N application under normal conditions, while the recovery rate of rice seedlings accelerated with the increase of N concentration after low temperature stress. These findings are similar to those of Liu et al. for the effect of N application on rice tillering after low temperature (Liu et al., 2019). They also found that N application under high intensity low temperature stress promoted tillering, but still could not make it return to normal levels. At day 12, the seedling heights of B116 N40 and N80 were close to CK, but B144 N80 still had a large gap with CK, which might be related to the poor cold stress tolerance of B144. There was still a large difference between the fresh and dry weights of the two rice varieties after stress compared with CK, indicating that N application cannot completely eliminate the adverse effects of the stress on rice seedlings. Some studies have shown that plant root growth and leaf photosynthesis are very sensitive to low temperature. The lack of nutrient uptake capacity and photosynthetic production capacity of rice seedlings may be an important reason for the difficulty of their rapid recovery in fresh and dry weight (Zhu et al., 2015; Yang et al., 2021). Humidity-cold combined stress will reduce the chlorophyll concentration and rubisco activity of rice seedlings, resulting in a decline in CO_2_ assimilation capacity (Wang et al., 2021b). It may require regulation in terms of photosynthesis above ground and nutrient uptake below ground and deserves further investigation, especially the combined application of phosphorus, potassium and N. Studies on other plants have shown that low light may promote an increase in plant seedling height (Huang et al., 2011; Lu et al., 2018). It has also been shown that plants under low light conditions increase leaf area and total chlorophyll to improve foliar light interception, but leaf thickness and lignin concentration show a decrease (Terashima and Hikosaka, 1995; Wang et al., 2022b). Weak light often causes a significant decrease in dry weight of crop seedlings (Supapohn et al., 2022), this may also be an important reason for the difficulty in the recovery of the fresh and dry weight for rice seedlings. Carbohydrates, especially sucrose and glucose, are the main signaling molecules affecting early plant seedling development. N is a major component of nucleic acids and proteins, and plays a key role in C transport and metabolism (Wang et al., 2021b; Li et al., 2022a). N effectiveness is a determining factor in the allocation of assimilated carbon in the synthesis of organic acids, starch and sucrose (Foyer et al., 2011). Enzymes involved in photosynthesis and nutrient metabolism are affected by a lack of light. Low light levels have a negative impact on yield, root vigor, carbohydrate accumulation, and leaf area index (Wei et al., 2018). Appropriate N application can increase the net photosynthetic rate of rice and compensate for the adverse effects caused by short-term low light (Pan et al., 2016). At day 12, the fresh and dry weights of B116 LN40 and B116 LN80 were close, but the seedling height of B116 LN80 was significantly higher than that of B116 LN40, indicating that excessive N concentration may lead to excessive rice seedling height and increase the risk of lodging. It also showed that biomass production is critical in determining yield variability in low temperature rice growing areas (Horai et al., 2014), so attention should be paid to the coordination of rice seedling height and robustness in production. In addition to N application, plant growth regulators should be applied in combination with reasonable plant growth regulators to coordinate plant height and stem thickness as well as above-ground and below-ground growth. This experiment was conducted only to simulate the late spring coldness weather commonly experienced by early rice in the middle and lower reaches of the Yangtze River of China. The low temperature level, light level, and duration of stress encountered in rice production may differ somewhat from this. The growth recovery of rice seedlings after experiencing lower average daily temperature and longer duration of low temperature still deserves further investigation. In addition, this study simulated “late spring coldness” with low temperature and weak light combined stress, and it did not design a single factor stress of low temperature and weak light. It is unknown about the stress effect for the single factor of low temperature and weak light on rice seedlings and the growth recovery efficiency of nitrogen, although it is generally believed that low temperature is the main harm factor.

ROS can integrate environmental stress signals, activate gene expression in response to stress, and thus brings about a balance between plant defense and growth (Wang et al., 2022c). Stress induces excessive production of ROS (including ${\text{O}}_{2}^{-}$ , H_2_O_2_ and OH^-^) in plants (Liu et al., 2018), resulting in oxidation and increased soluble protein concentrations, which is beneficial in enhancing plant tolerance to stress, but can also have an impact on normal plant physiological processes (Yang et al., 2014; Fanourakis et al., 2020). Antioxidant enzymes (e.g. SOD, POD, CAT) are able to scavenge excess ROS. This study showed that the stress increased the soluble protein content as well as the MDA content of the two rice varieties. N application increased the soluble protein level and rapidly reduced the H_2_O_2_ and MDA content, while the antioxidant enzyme activity of rice seedlings was increased and the stress damage to rice seedlings was reduced. It has been suggested that high N application can improve plant stress tolerance by enhancing antioxidant capacity and inhibiting lipid peroxidation (Zhong et al., 2017), and the results of Liu et al. similarly confirmed this idea (Liu et al., 2019; Liu et al., 2022). The LN20, LN40, and LN80 of both the two varieties showed higher soluble protein content on day 3 than on day 0 instead of decreasing rapidly, presumably due to the high uptake of nitrogen by rice seedlings in the short term after rewarming, resulting in an increase in soluble protein content (Reguera et al., 2013; Huang et al., 2021). Meanwhile, comparing B116 with B144, it could be found that the antioxidant enzyme activity of B116 was higher than that of B144 in most cases with or without N application. This result could be mutually confirmed with the consistently lower MDA level of B116 than that of B144, showing the variability of physiological response after the stress in different genotypes.

Nitrogen is one of the most important nutrients for plants, and low temperature hinders the uptake of nitrogen by plants mainly by causing damage to cell membranes (Shimono et al., 2012; Waraich et al., 2012). In contrast, high N application rates under low temperature conditions can promote nitrogen uptake, tillering, and foliar development in rice (Zhou et al., 2018). ${\text{NO}}_{3}^{-}$ and ${\text{NH}}_{4}^{+}$ are absorbed and converted by the nitrate peptide transporter family/transporters (NPF/NRTs) protein family and ammonium transporters (AMTs) protein family in plants, respectively. Most of ${\text{NO}}_{3}^{-}$ is transported to ammonium nitrogen by NR and NiR in plants, which is further assimilated to organic form by GS/GOAT for the biosynthesis of nitrogenous compounds in plants (Xing et al., 2022). Nhd1 can directly bind to the promoters of *OsAMT1.3* and *OsNRT2.4*, activating their expression and regulating the uptake and partitioning of ${\text{NH}}_{4}^{+}$ and ${\text{NO}}_{3}^{-}$ in rice, thus affecting the uptake and utilization of N in rice (Li et al., 2022b). The present study showed that low temperature significantly inhibited the uptake of nitrogen by both rice seedlings. Along with N application after low temperature stress, the expression of N uptake and transport-related genes in rice seedlings was significantly increased, while the activities of NR and GS were significantly enhanced, and N accumulation was significantly increased. This indicates that increasing N application after low temperature and weak light stress can promote N uptake by rice seedlings, which in turn ensures the nutrients required for rapid growth recovery. Liu found that rice with normal N application after low temperature could accumulate N at the 10.5 leaf stage close to or even higher than rice at the control temperature (Liu et al., 2019), However, our results showed that there was still a large difference in N accumulation between LN0 and LN20 at 12 days after low temperature compared with CK. It is conjectured that the difference in treatment temperature and treatment period may have led to different damage caused by low temperature to rice seedlings, while the investigation of rice growth in this study only lasted until day 12 and no subsequent investigation was conducted. In addition, we found in our previous study that the stress caused different degrees of prolongation of the fertility of B116 and B144. Therefore, appropriate increase of N application in low temperature years is extremely important for growth recovery of rice, and further promotion of late reproductive differentiation remains an issue of concern.

Plant growth and development as well as adaptation to adversity are two contradictory processes (Scheres and van der Putten, 2017). The endogenous hormones are important for the synergistic regulation of plant growth and development, adaptation, and resistance to external stresses (Wuddineh et al., 2015; Waadt et al., 2022). Stress can induce ABA and H_2_O_2_ production, which can act as signaling molecules involved in plant response to adversity (Ye et al., 2011). ABA, as the most important “stress hormone”, is not only related to plant tolerance to stress, but also has a role in inhibiting seed germination, shaping leaf morphology, and inhibiting cell growth (Hwang et al., 2010; Wang et al., 2021a; Gao et al., 2022), while gibberellin has the effect of accelerating cell elongation and regulating plant height (Sasaki et al., 2002). N has been shown to be involved in the synthesis and transport of ABA and gibberellin in plants. Among them, ABA biosynthesis is positively correlated with high concentrations of ${\text{NO}}_{3}^{-}$ in the roots (Signora et al., 2001). ${\text{NO}}_{3}^{-}$ supply can increase gibberellin synthesis by promoting DELLA degradation (Camut et al., 2021), and low concentrations of ${\text{NO}}_{3}^{-}$ can down-regulate gibberellin biosynthetic genes in maize, up-regulate GA catabolism and thus reduce the level of bioactive gibberellins in maize roots (Wang et al, 2020b). Our study showed that after the stress, the GA_3_ content of B116 decreased and the ABA content increased, and the GA_3_/ABA ratio decreased substantially. Under cold stress, plants can regulate the ABA/GA_3_ balance through ABA signaling to resist stress (Wang et al., 2021b). After the stress, the ABA content of B144 showed a small decrease compared with CK, and the GA_3_/ABA ratio also decreased much less than that of B116 compared with CK, which is consistent with the previous performance that B144 has a weak antioxidant capacity. In the later stage, N can increase GA_3_/ABA ratio by increasing GA_3_ content and decreasing ABA content, thus promoting rapid growth recovery of rice seedlings. RT-qPCR assay showed that the stress suppressed gibberellin biosynthesis and ABA catabolism in both rice seedlings, while promoting gibberellin catabolism and ABA biosynthesis as a way to maintain a low GA_3_/ABA ratio to withstand the stress. N application could promote gibberellin biosynthesis and ABA catabolism, while inhibiting gibberellin catabolism and ABA biosynthesis in the late stage, allowing rice seedlings to maintain a high GA_3_/ABA ratio and recover growth rapidly. Furthermore, we will use transcriptomic, proteomic and metabolomic analyses to identify specific biological pathways, as will help us to gain a deeper and more comprehensive understanding for the mechanisms of nitrogen application accelerates rapid growth and recovery of rice seedlings after cold stress.

## 5 Conclusion

**Figure 8:**
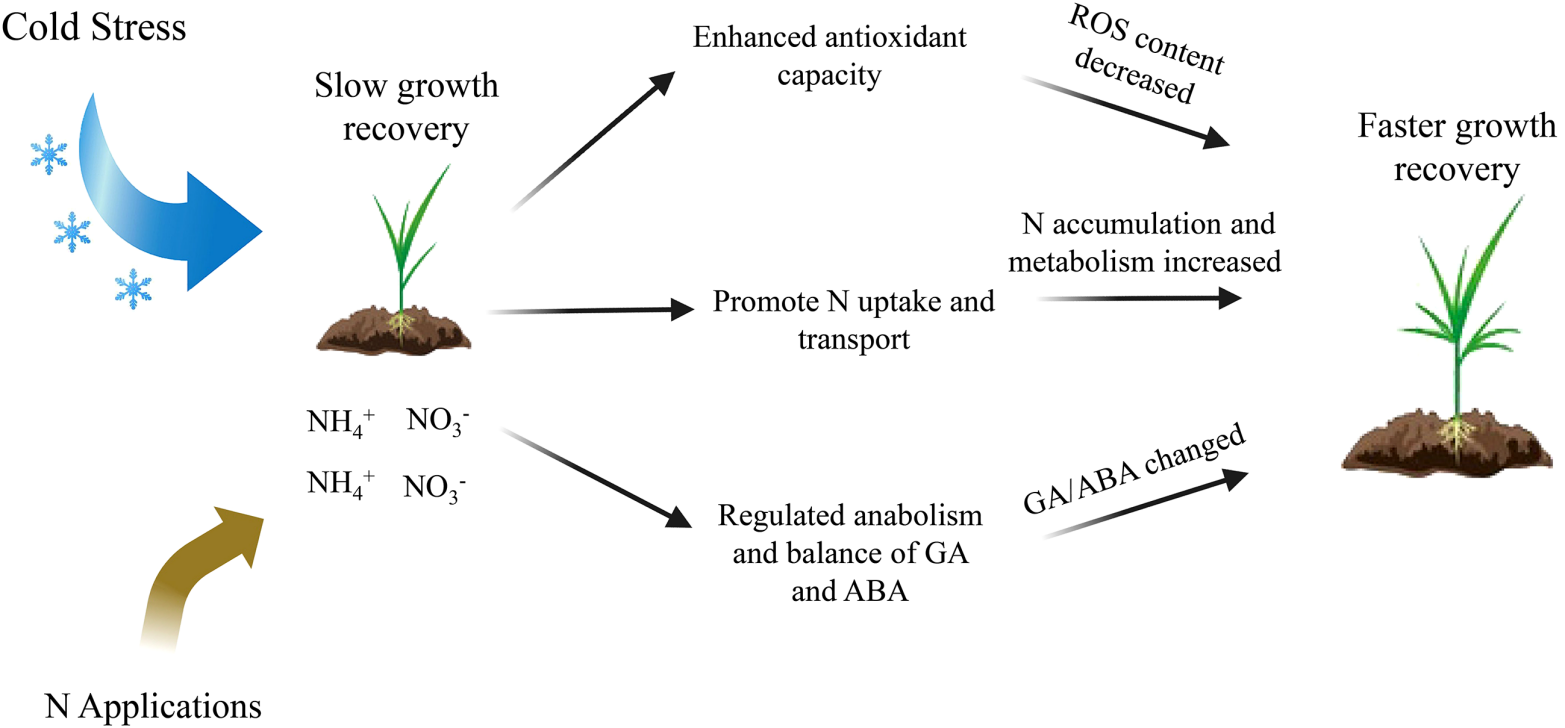
Working model of the role of N in the growth recovery of rice seedlings after low temperature and weak light stress. N enhances the antioxidant capacity of rice seedlings after the stress, improves their nitrogen metabolism, and regulates the biosynthesis and metabolism of gibberellin and ABA in their vivo, thus enabling seedlings to recover growth rapidly.

Low temperature and weak light stress tend to cause ROS accumulation and membrane lipid peroxidation in rice seedlings, resulting in cell damage. After stress, rice seedlings often show slow growth. Timely application of N could regulate the nitrogen metabolism and active oxygen scavenging enzyme activity and hormone levels of rice seedlings, helping it to recover seedling height as well as biomass. At day 12, the seedling height, fresh weights and dry weights in B116 LN40 improved by 22.1%, 41.8% and 47.4% compared to B116 LN0, while improved by 21.1%, 46.9% and 48.6% in B144 LN40 compared to B144 LN0, respectively. However, the N application group was still significantly lower than the CK group in terms of fresh weight and dry weight. The fresh and dry weights of B116 LN40 reached 72.8% and 64.3% of the CK group, while those of B144 LN40 t only reached 61.7% and 37.2%, respectively. How to further enhance the photosynthetic production capacity of leaves and maintain the balance between stem fullness and plant height of rice seedlings through fertilization as well as application of exogenous growth regulators deserves deeper study. This study also shows that varieties with weak growth recovery ability were more difficult to recover growth indicators after stress, so attention should be paid to the selection of rice varieties with strong growth recovery ability after low temperature and weak light when growing in the field. Applying the appropriate amount of N after low temperature can increase the activity of antioxidant enzymes including SOD, POD and CAT in rice seedlings, which helps to reduce the ROS damage of the stress on rice seedlings. Higher N uptake and transport capacity, as well as higher NR, GS activity were beneficial to enhance the N metabolism of rice seedlings and promote N accumulation in rice seedlings after the stress. Meanwhile, N application was able to regulate the synthesis and metabolism of gibberelin and ABA in rice seedlings, and then regulate the balance of the two hormones after stress. The GA_3_/ABA ratio of rice seedlings was maintained at an appropriate level, which maintained the resistance of rice seedlings in the early stage (day 0 to day 6) and at higher level helped rice seedlings recover rapidly in the later stage (day 6 to day 12) (**Figure 8**). The results of this study will provide a reference for the regulation of N on the recovery of early rice seedling growth after low temperature and weak light stress. In particular, it is of practical reference value for the formulation of remedial measures when early rice direct seeding encounters late spring coldness in southern China.

## Data availability statement

The original contributions presented in the study are included in the article/**Supplementary Material**. Further inquiries can be directed to the corresponding author.

## Author contributions

XC conceptualized and designed the experiment; HW and LZ, participated in all experimental procedures and drafted the manuscript. XF, and SH performed data analysis and participated in figure preparation. DZ participated in sample preparation and the measurement of multiple indicators. XC and HH revised the manuscript. All have read and agreed to the published version of the manuscript.

## Footnotes

1 Standardization Administration of China, 2017. National Standard of the People’s Republic of China for Inversion Weather Indicators. http://www.gb688.cn/bzgk/gb/newGbInfo?hcno=A9917F534E4F8CC0C49BB62591E3C6E5 (accessed on 30 July 2022)
