# Supplementary material for: Physiological analysis reveals the mechanism of accelerated growth recovery for rice seedlings by nitrogen application after low temperature stress
Source: Front Plant Sci. 2023 Feb 16;14:1133592. doi: 10.3389/fpls.2023.1133592 (PMC9978396; doi:10.3389/fpls.2023.1133592)
Supplement: Supplementary file 1 [file DataSheet_1.pdf]

Supplementary Material

1 Supplementary Figures and Tables

1.1 Supplementary Figures

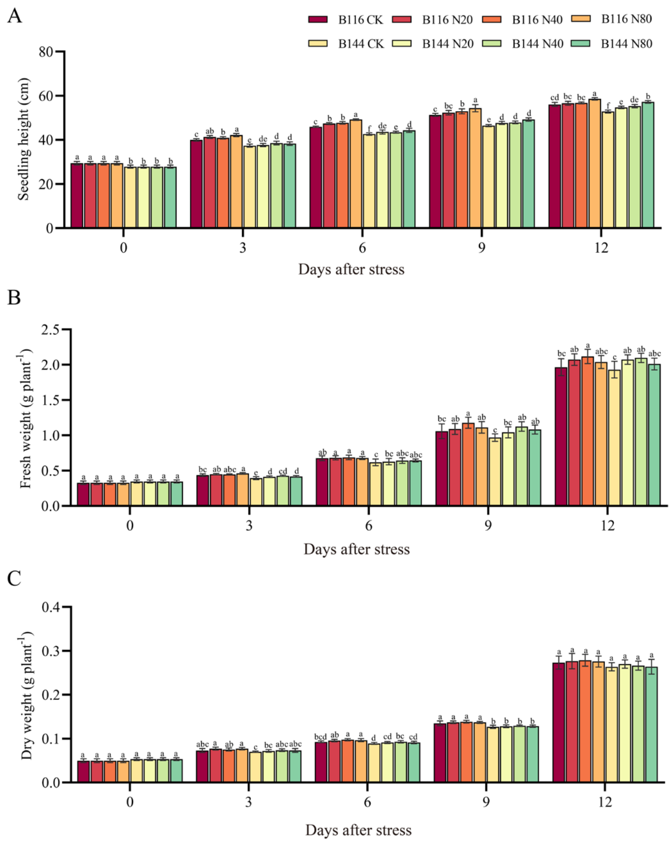

Supplementary Figure 1. Comparison of the differences in seedling height (A), fresh weight (B) and dry weight (C) of rice seedlings at different periods after N application in the control environment. CK, N20, N40, N80 represent 0 kg hm^{-2}, 20 kg hm^{-2}, 40 kg hm^{-2} and 80 kg hm^{-2} urea

application, respectively. Values are means ± standard deviations of three biological replicates, and different lowercase letters represent significant differences at p<0.05 based on Duncan's test.

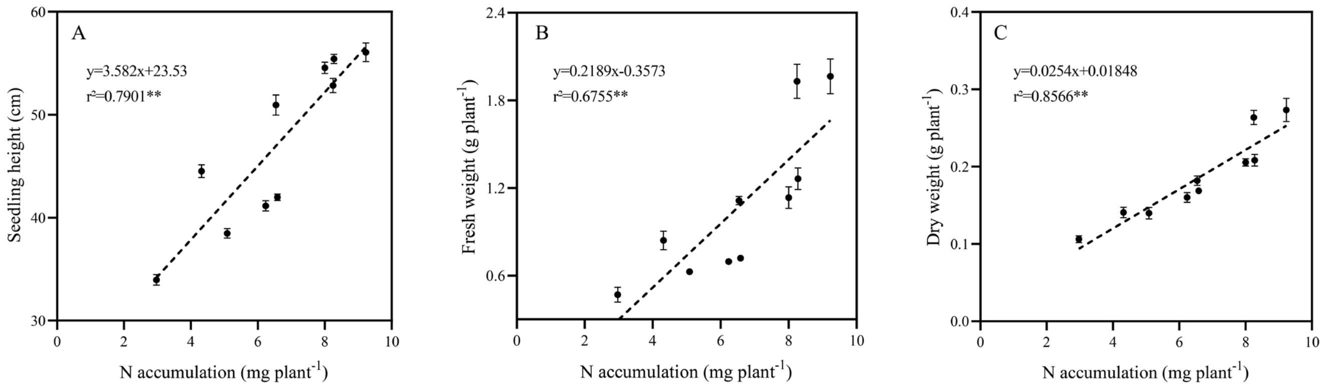

Supplementary Figure 2. Relationship between rice N accumulation and rice seedling height (A), fresh weight (B) and dry weight (C) increment after 12 days of N application after low temperature and weak light treatment.

1.2 Supplementary Table

Supplementary Table 1. List of qRT-PCR primers used.

Primer	Sequence (5' to 3')	Base number
Os01g0883800-F	TTCATGGCGCTGTCGAACGG	20
Os01g0883800-R	CCAGGTGAAGTCCGGGTAGT	20
Os06g0570100-F	CTTTACCAAGAGATCCGGGAGGT	23
Os06g0570100-R	AGGTTGATCACCATCTCGGTGC	22
Os02g0630300-F	TTCAGGCGTGGAGCAACAAC	20
Os02g0630300-R	GTACTCCCCGAAGGTGAACGC	21
Os03g0645900-F	TGCTCGACAAGGAGAAGACGTCG	23
Os03g0645900-R	ACTCGTTGAAGATGGAGTCGGC	22
Os04g0448900-F	GACCGTTAAGCTGTAGGCTTTCTG	24
Os04g0448900-R	ATCTGACCGGCTTCCAATGG	20
Os08g0472800-F	CCCGGACTACTTCCAGGATCC	21
Os08g0472800-R	CGACGATCTCCCACCTGTAGG	21
Os08g0157600-F	CCAACACACCGTCAAGTAGTGAT	23
Os08g0157600-R	AAAGCCAGACGACCCTCTTCA	21
Os04g0509600-F	ATCATCTGCGGGTTCGTCTCG	21
Os04g0509600-R	GCCGTAGATCTCCTCGACGTACT	23
Os02g0620500-F	TGACGACGCTCTACGGCAAGA	21
Os02g0620500-R	ACAGCTTGTTGCAGCCGATGA	21

Os01g0547600-F	CCTTCGTCTGCAAAAGGTCGC	21
Os01g0547600-R	CCGGAAATGGATGAGGGCGA	20
OsActin-F	GGCCAACAGGGAGAAGATGACAC	23
OsActin-R	GATCCCTACCAGCAAGATCAAGACG	25

Supplementary Table 2. Results of two-way ANOVA on differences in seedling height, fresh weight and dry weight of rice under normal conditions.

Factors	Seedling height	Fresh weight	Dry weight
Variety (V)	< 0.001	< 0.01	< 0.001
Nitrogen (N)	< 0.001	< 0.001	0.141
Growth recovery time (T)	< 0.001	< 0.001	< 0.001
V*N	ns	ns	ns
V*T	**	*	**
N*T	**	**	ns
V*N*T	*	ns	ns

* and ** represent $P < 0.05$ and $P < 0.01$, respectively, ns represents no significant.

Supplementary Table 3. Results of two-way ANOVA on the differences in soluble protein content, MDA content and antioxidant enzyme activities in rice after the stress.

Factors	Soluble protein content	MDA content	SOD activity	POD activity	CAT activity	H_2O_2 content
Variety (V)	< 0.001	< 0.001	< 0.001	< 0.001	< 0.001	< 0.001
Nitrogen (N)	< 0.001	< 0.001	< 0.001	< 0.001	< 0.001	< 0.001
Growth recovery time (T)	< 0.001	< 0.001	< 0.001	< 0.001	< 0.001	< 0.001
V*N	ns	ns	**	ns	ns	ns
V*T	**	**	**	**	**	ns
N*T	**	**	**	**	**	ns
V*N*T	ns	ns	**	**	**	ns

Supplementary Table 4. Results of two-way ANOVA on the differences in NR，GS activities and N accumulation in rice after the stress.

Factors	NR activity	GS activity	N accumulation
Variety (V)	< 0.001	< 0.001	< 0.001
Nitrogen (N)	< 0.001	< 0.001	< 0.001
Growth recovery time (T)	< 0.001	< 0.001	NA
V*N	**	ns	ns
V*T	**	**	NA
N*T	**	**	NA
V*N*T	**	**	NA

Supplementary Table 5. Results of two-way ANOVA on the differences in GA_3 and ABA content in rice after the stress.

Factors	GA_3 content	ABA content
Variety (V)	< 0.001	< 0.001
Nitrogen (N)	< 0.001	< 0.001
Growth recovery time (T)	< 0.001	< 0.001
V*N	**	**
V*T	**	**
N*T	**	**
V*N*T	**	**
